# New quaternary half-metallic ferromagnets with large Curie temperatures

**DOI:** 10.1038/s41598-017-01782-5

**Published:** 2017-05-11

**Authors:** Ashis Kundu, Srikrishna Ghosh, Rudra Banerjee, Subhradip Ghosh, Biplab Sanyal

**Affiliations:** 1 Department of Physics, Indian Institute of Technology Guwahati, Guwahati, 781039 Assam India; 2 Department of Physics and Astronomy, Uppsala University, Box 516, 75120 Uppsala, Sweden

## Abstract

New magnetic materials with high Curie temperatures for spintronic applications are perpetually sought for. In this paper, we present an ab initio study of the structural, electronic and magnetic properties of Quaternary Heusler compounds CoX′Y′Si where X′ is a transition metal with 4*d* electrons and Y′ is either Fe or Mn. We find five new half-metallic ferromagnets with spin polarisation nearly 100% with very high Curie temperatures. The variation of Curie temperatures as a function of valence electrons can be understood from the calculated inter-atomic exchange interaction parameters. We also identify a few other compounds, which could be potential half-metals with suitable application of pressure or with controlled doping. Our results reveal that the half-metallicity in these compounds is intricately related to the arrangements of the magnetic atoms in the Heusler lattice and hence, the interatomic exchange interactions between the moments. The trends in the atomic arrangements, total and local magnetic moments, interatomic magnetic exchange interactions and Curie temperatures are discussed with fundamental insights.

## Introduction

The study of intermetallic Heusler compounds has gained significant momentum over the last decade due to their exceptional qualities in displaying wide varieties of properties, ranging from half-metallic magnetism, magnetic shape memory effect, spin gapless semiconductor to giant magnetocaloric effect, thermoelectric effect and superconductivity^1–6^. These properties have been exploited successfully for technological applications. For example, the half-metallicity in ferromagnetic materials is being used for spintronics applications such as spin injectors^7^, magnetic tunnel junctions^8^, spin valves^9^ and spin torque transfer-random access memories^10^; the zero gap trait in spin gapless semiconductors is being used for studying spin Seebeck effect^11^.

The largest number of magnetic Heusler compounds investigated are ternary intermetallics X_2_Y′Z where X and Y′ are transition metals and Z is a main group element. It is apparent that the novel magnetic properties in these compounds arise due to the presence of unfilled *d* shell of more than one transition metal components. An intricate relationship between the occupancies of the magnetic constituents in sub-lattices with different symmetries, the magnetic structures associated and the electronic structures resulting due to these two, have been found to be responsible behind the emergence of the novel phenomena observed in these materials. If each sub-lattice, in a Heusler structure, is occupied by a different element, resulting in a XX′Y′Z compound, with X′ a magnetic element different than X, then there will be more sub-classes (depending on the sub-lattice occupancies of the three magnetic elements) of the structure than that possible with X_2_Y′Z compounds. Consequently the quaternary Heusler compounds present themselves as a class of materials where greater flexibilities with the choices of components and their arrangements on lattice sites may offer discovery of newer materials with target properties. Keeping this in mind, research on quaternary Heusler compounds has started in recent times. Quite a few compounds in the quaternary Heusler family have turned out to be promising for spintronics based applications displaying half-metallicity^12, 13^, high Curie temperature^14^ and spin gapless semiconducting properties^15, 16^.

An overwhelming majority of the ternary and quaternary compounds, observed to be exhibiting properties useful for spintronics applications, have the magnetic atoms with unfilled 3*d* shells. Only a handful have a combination of elements from the series of 3*d* and 4*d* elements in the periodic table^17–22^. The presence of both 3*d* and 4*d* electrons in the same compound should present interesting perspectives in comparison with systems having only 3*d* electrons in their outer shells. However, to our knowledge, there has been no systemic investigation of a series of quaternary Heusler compounds having 3*d* and 4*d* electron constituent. In this paper, we, therefore, have explored two quaternary Heusler series CoX′MnSi and CoX′FeSi, where X′ is varied from Y to Ag along the series in the periodic table. The reason behind picking up CoX′Y′Si systems was the following: Co_2_Y′Z ternary Heusler compounds have turned out to be half-metallic magnets with high Curie temperatures^23^ and are therefore useful for spintronics applications. It would be interesting to explore whether new half-metallic magnets with high Curie temperatures can be derived from the family of Heusler compounds by changing the compositions and thus expand the database of materials for spintronics applications. Apart from the quest for new materials, our motivations were (1) to understand the effects of the presence of an element with 4*d* electrons on the structure, electronic and magnetic properties of compounds where the other two magnetic elements are from 3*d* transition metal series and (2) To understand the role of the 4*d* transition metal elements by successively changing it along the series of 4*d* transition metals so that the total electron number changes continuously, on the properties of compounds where one of the 3*d* elements remains fixed while the other changes.

To this end, we have used Density functional theory (DFT) based electronic structure method to compute the electronic structures, the structural, electronic and magnetic properties of quaternary CoX′MnSi and CoX′FeSi compounds where X′ varies from Y to Ag. We have specifically explored the ground state sub-lattice occupancies and the magnetic structures of each compound. In subsection II, computational details are given. In the following, we present and discuss our results followed by a conclusion and future outlook. The methods used in this work are given at the end.

## Results

### Structural Properties

The Heusler compounds with X_2_Y′Z composition crystallize in the cubic L2_1_ (Space group no. 225; $Fm\bar{3}m$) structure where the X atoms occupy the Wyckoff position 8c (1/4, 1/4, 1/4), the Y′ and Z atoms occupy the positions 4b(1/2, 1/2, 1/2) and 4a(0, 0, 0) respectively. The Y′ and Z atoms are typically the least and the most electronegative elements, and thus form a rocksalt (NaCl) type lattice. Due to the ionic character of their ineractions, they coordinate octahedrally. The X atoms, with electronegativities in between those of Y′ and Z, fill the tetrahedral voids^23^. The structure can also be understood as a zinc-blende type sub-lattice, build up by one X and Z, having covalent bonding between them, the other X occupying the tetrahedral holes while the Y′ occupying the octahedral holes^24^.

In addition to this, an inverse Heusler structure, the Hg_2_CuTi (space group no. 216; $F\bar{4}3m$) structure, can be observed in the Heusler family. In case of the inverse Heusler structure, the atom at 4b site gets interchanged with one of the atoms at 8c site of regular Heusler alloy. Thus, the X atoms of X_2_Y′Z compounds occupy the Wyckoff positions 4c(3/4, 3/4, 3/4) and 4b(1/2, 1/2, 1/2) whereas the Y′ and the Z atoms occupy the 4c(1/4, 1/4, 1/4) and 4a(0, 0, 0) positions respectively^25^. The system crystallizes in this structure when the number of valence electrons of the transition metal element Y′ is smaller than the that of the transition metal element X. Experimental and theoretical studies on numerous Heusler compounds established the above rules for site occupancies of the ground states of X_2_Y′Z Heusler compounds^25^.

**Figure 1:**
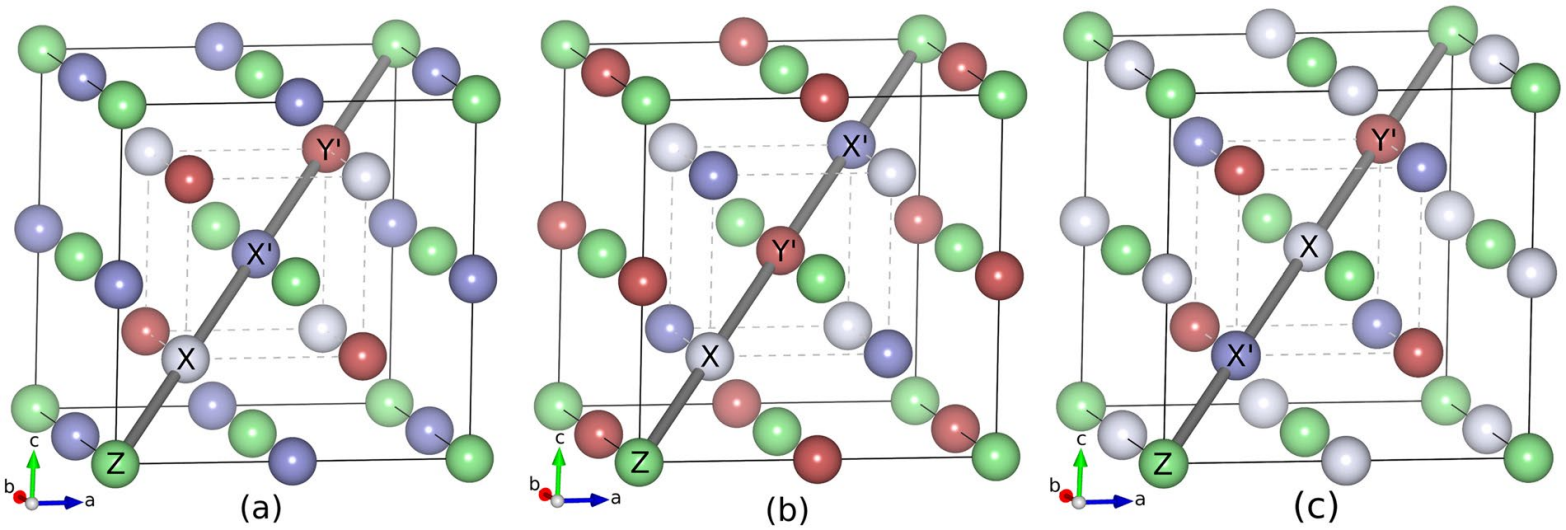
Crystal structure of XX′Y′Z in (**a**) Type-I (X and Y′ are in symmetric positions) (**b**) Type-II (X and X′ are in symmetric positions) (**c**) Type-III (Y′ and X′ are in symmetric positions).

When one of the X atoms in X_2_Y′Z compound is substituted by a different transition metal element X′, a quaternary Heusler alloy (QHA) with composition XX′Y′Z is formed. The prototype structure of QHA is LiMgPdSn (Space group no. 216; $F\bar{4}3m$)^26^ with X, X′ and Y′ occupying 4c, 4d and 4b positions respectively, making the arrangement of atoms along the body diagonal of the Heusler lattice as X-Y′-X′-Z. It has been found out that if the number of valence electrons decreases along X, X′, Y′ then LiMgPdSn structure is the most stable^3^. However, there are other two inequivalent configurations possible if one fixes the position of Z as the site 4a and permutes the occupancies of the three other sites. The three resulting configurations^3, 13, 27^ are shown in Table 1. In order to find the ground state crystal structures of the CoX′Y′Z compounds considered in the present study, we have calculated the total energies of each of the compounds in all three structure types listed in Table 1. The results are shown in Table 2. Our calculations show that the structure Type-III is energetically always higher than the other two structure types for all the compounds considered. Thus, the compounds crystallize either in structure Type-I (T_*I*_) or in Type-II (T_*II*_) (Fig. 1). It may be noted that the order of magnitude of energy differences (Δ*E*, Δ*E*′) is consistent with those obtained for quaternary Heusler alloys with 3*d* magnetic atoms only^12^. The results also suggest that the argument with respect to the number of valence electrons of each transition metal atom, in explaining the structure type in which a particular compound crystallizes to, is not valid for all the compounds studied. According to that argument, CoMoMnSi, CoTcFeSi and CoMoFeSi should have crystallized in Type-I structures while CoAgMnSi and CoAgFeSi should have crystallized in Type-II structures.

**Table 1:** Possible structure types of XX′Y′Z compounds.

	4a (0, 0, 0)	4b (1/2, 1/2, 1/2)	4c (1/4, 1/4, 1/4)	4d (3/4, 3/4, 3/4)
Type-I (T_I_)	Z	X′	X	Y′
Type-II (T_II_)	Z	Y′	X	X′
Type-III (T_III_)	Z	X	X′	Y′

**Table 2:** Calculated lattice constants, formation energies, magnetic moments and energy differences between possible structures of quaternary CoX′Y′Si compounds.

Systems (Type-I)	Lattice constant (Å)	Formation energy (eV)	Δ*E* (eV/f.u.)	Δ*E*′ (eV/f.u.)	Structure type	M (*μ*_*B*_/*f*.*u*.)
CoYMnSi	6.30	−1.94	0.39	0.05	T_I_	4.55
CoZrMnSi	5.96	−2.23	1.14	0.82	T_I_	0.00
CoNbMnSi	5.85	−1.74	0.69	0.85	T_I_	1.00
CoMoMnSi	5.84	−1.90	−0.21	0.67	T_II_	2.33
CoTcMnSi	5.79	−3.14	−0.89	0.84	T_II_	3.00
CoRuMnSi	5.79	−2.70	−1.38	0.85	T_II_	4.00
CoRhMnSi	5.83 (5.84^49^)	−2.82	−1.46	0.81	T_II_	5.00 (5.00^49^)
CoPdMnSi	5.90	−2.06	−0.67	0.51	T_II_	4.90
CoAgMnSi	5.96	−0.38	0.05	0.28	T_I_	3.53
CoYFeSi	6.08	−2.14	0.91	0.81	T_I_	0.00
CoZrFeSi	5.96 (5.97^21^)	−3.33	1.19	1.24	T_I_	1.00 (1.00^21^)
CoNbFeSi	5.88	−2.31	0.77	1.06	T_I_	1.99
CoMoFeSi	5.84	−1.42	−0.05	0.39	T_II_	2.83
CoTcFeSi	5.80	−2.65	−0.67	0.49	T_II_	3.89
CoRuFeSi	5.78 (5.773^14^)	−3.12	−1.02	0.52	T_II_	4.66 (4.80^14^)
CoRhFeSi	5.80	−2.10	−0.78	0.21	T_II_	4.90
CoPdFeSi	5.86	−1.48	−0.23	0.06	T_II_	4.00
CoAgFeSi	5.88	−0.10	0.09	0.18	T_I_	2.71

The energy differences between Type-II (T_*II*_) and Type-I (T_*I*_) structures are given by ΔE = ${{\rm{E}}}_{{{\rm{T}}}_{{\rm{II}}}}$ − ${{\rm{E}}}_{{{\rm{T}}}_{{\rm{I}}}}$. A positive ΔE implies T_*I*_ as the ground state. The energy differences between Type-III (T_*III*_) and Type-I or Type-II structures are given by ΔE′ = ${{\rm{E}}}_{{{\rm{T}}}_{{\rm{III}}}}$ − ${{\rm{E}}}_{{{\rm{T}}}_{{\rm{I}}}/{{\rm{T}}}_{{\rm{II}}}}$. For the systems which crystallise in Type-_II_ (T_II_), ΔE′ = ${{\rm{E}}}_{{{\rm{T}}}_{{\rm{III}}}}$ − ${{\rm{E}}}_{{{\rm{T}}}_{{\rm{II}}}}$; for the systems which crystallise in Type-I (T_I_), ΔE′ = ${{\rm{E}}}_{{{\rm{T}}}_{{\rm{III}}}}$ − ${{\rm{E}}}_{{{\rm{T}}}_{{\rm{I}}}}$.

**Figure 2:**
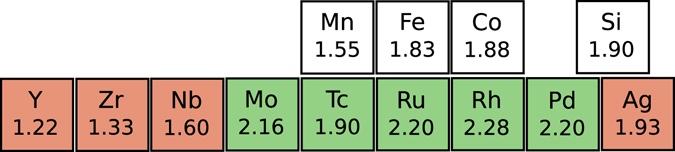
Schematic representation of preferred ground state structures (Red - Type-I; Green - Type-II) of CoX′Y′Si compounds. The number attached to each element represent it’s electronegativity (Pauli scale).

A better understanding of the site occupancies displayed by these compounds can be achieved if one provides an argument only in terms of relative electronegativities of the transition metal components in these systems. If one fixes the position of the main group element at 4a site, then the 4b site should be occupied by the least electronegative element out of the three transition metal atoms. Thus the main group element and the least electronegative element of the remaining three occupy the octahedral sites while the other two occupy the tetrahedral sites. For example, in CoMoMnSi, Mn is the least electronegative among the three transition metal atoms and so it occupies the 4b site, giving rise to a Type-II structure. The reason behind unfavourable Type-III structure for all compounds, too, can be extracted from this logic. In Type-III structure, Co and Si, the two elements having the highest electronegativities occupy the octahedral sites, thus making it impossible to form the rocksalt sub-lattice with octahedral coordination. However, the exceptions to this empirical rule are observed in cases of CoNbMnSi, CoAgMnSi and CoAgFeSi. The reason behind the exception in case of CoNbMnSi could be due to the fact that the electronegativities of Nb and Mn are very close. In cases of CoAgMnSi and CoAgFeSi, the energy differences between Type-I and Type-II structures are very small (Table 2), indicating that the structure Type-I is not strongly preferred over structure Type-II and a thermally induced disordered structure can be the ground state for them. Nevertheless, the site preferences for a large number of QHA can be explained by this suggested empirical rule^13, 15, 24, 27–36^. A schematic representation of preferred structures of CoX′Y′Si compounds under consideration is given in Fig. 2.

The optimized lattice constants in the ground state structures of CoX′Y′Si systems and their formation energies are also given in Table 2. The variations in the lattice constants with the element X′ are consistent with the variations in the atomic radii of X′ for both the series. The formation energies are expressed as

$${E}_{f}={E}_{Co{{\rm{X}}}^{^{\prime} }{{\rm{Y}}}^{^{\prime} }Si}-({E}_{Co}+{E}_{{{\rm{X}}}^{^{\prime} }}+{E}_{{{\rm{Y}}}^{^{\prime} }}+{E}_{Si})$$

where *E*_*Co*X′Y′*Si*_ is the total energy of the CoX′Y′Si per formula unit and *E*_*Co*_, *E*_X′_, *E*_Y′_, and *E*_*Si*_ are the total energies of the bulk Co, X′, Y′ and Si respectively in their ground state structures. The results given in Table 2 illustrate that all compounds are probable to form. Out of these compounds, only CoRuFeSi has been experimentally synthesised^14^. Our calculated ground state structure and the lattice constant for this system agree very well with the experimental result. The negative values of formation energies for all compounds suggest that all of them are stable from the point of view of enthalpy and therefore further investigations into their properties are worth a shot.

#### The Magnetic moments and the Slater-Pauling Rule

**Figure 3:**
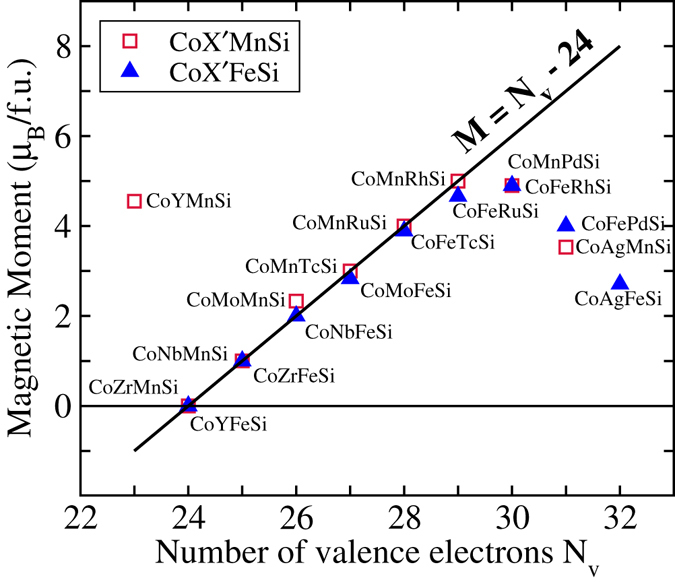
Total magnetic moments versus the total number of valence electrons *N*_*ν*_ for CoX′Y′Si compounds. The Slater-Pauling *M* = *N*_*ν*_ − 24 line is drawn as a guide to understand whether the compounds follow Slater-Pauling rule.

The necessary condition for half-metallic behaviour in Heusler compounds is that the total magnetic moment per formula unit is an integer and that it follows the Slater-Pauling rule^37, 38^, which connects the total magnetic moment to the number of valence electrons. For X_2_Y′Z Heusler alloys, the magnetization *M* and the number of valence electrons *N*_*ν*_ are related either by *M* = *N*_*ν*_ − 18 or by *M* = *N*_*ν*_ − 24 or by *M* = *N*_*ν*_ − 28, depending on whether X is an early transition metal or not^1, 39^. It was observed that half-metallic quaternary Heusler compounds too obey these rules^3^. Since CoX′Y′Si systems are derived from Co_2_Y′Si (Y′ = Mn, Fe) compounds which follow *M* = *N*_*ν*_ − 24 Slater-Pauling rule^1^, we intend to investigate whether the systems under study in the present work conform to that. Accordingly, we have plotted total magnetic moments (Table 2) of all compounds in Fig. 3 along with *M* = *N*_*ν*_ − 24 line. We find that the magnetic moments for most of the compounds in CoX′Y′Si series lie on or very close to the *M* = *N*_*ν*_ − 24 line. The significant deviants from the Slater-Pauling line are the ones where X′ element is a late transition metal. The magnetic moments of most of these deviants, shown in Table 2, actually do not seem to follow any of the Slater-Pauling rules mentioned above and thus are not interesting from the perspective of half-metallic magnets. CoYMnSi, in spite of deviating from the Slater-Pauling line *M* = *N*_*ν*_ − 24 significantly (*M* should have been −1 *μ*_B_ according to *N*_*ν*_ − 24 rule) comes close to following *M* = *N*_*ν*_ − 18. CoPdFeSi, on the other hand, has an integer moment, yet does not fit into any of the Slater-Pauling rules.

The results on magnetic moments, thus, suggest that only six compounds (CoNbMnSi, CoTcMnSi, CoRhMnSi, CoRuMnSi, CoZrFeSi, CoNbFeSi) with non-zero magnetic moments follow the *M* = *N*_*ν*_ − 24 Slater-Pauling rule and are, therefore, potential half-metallic magnets. In the next sub-section, we discuss their electronic structures to confirm whether they indeed can be considered as new half-metallic magnets.

### Electronic Structure

The origin of the half-metallic gap in Co_2_Y′Z compounds has been understood from the features in their electronic structures near the Fermi level. In these compounds, a gap in the spin down bands arise due to the hybridisations between the *d* orbitals of Co and Y′. The *d* orbitals of the Co atoms hybridise and create 5 bonding hybrids (2 e_*g*_ and 3 t_2*g*_) which further hybridise with the d orbitals of the Y′ element resulting in 5 bonding and 5 anti-bonding hybrids. Five non bonding hybrids (2 e_*u*_ and 3 t_1*u*_) of the octahedral Co atoms can not hybridise with the *d* orbitals of tetrahedral Y′ atoms. The gap in the spin down bands arise from these non bonding states^1^. For most of half-metallic Co_2_Y′Z compounds, the half-metallic gaps are due to the separations between the bottom of the conduction bands consisting of e_*u*_ states and the top of the valence bands made up of non bonding t_1*u*_ hybrids. This pattern of hybridisation is largely followed in case of X_2_Y′Z compounds (X, Y′ are 3*d* transition metals) having Inverse Heusler arrangement whose magnetisations follow either *M* = *N*_*ν*_ − 18 or *M* = *N*_*ν*_ − 24 Slater-Pauling rules^39^. Since the structure Type-I and Type-II in the present study are the quaternary counterparts of the ternary X_2_Y′Z Inverse Heusler and regular Heusler compounds with one X atom replaced with an element from 4*d* series, respectively, it would be interesting to explore how the hybridisation picture in case of X_2_Y′Z with all magnetic atoms from 3*d* series gets modified for the systems presented here.

**Figure 4:**
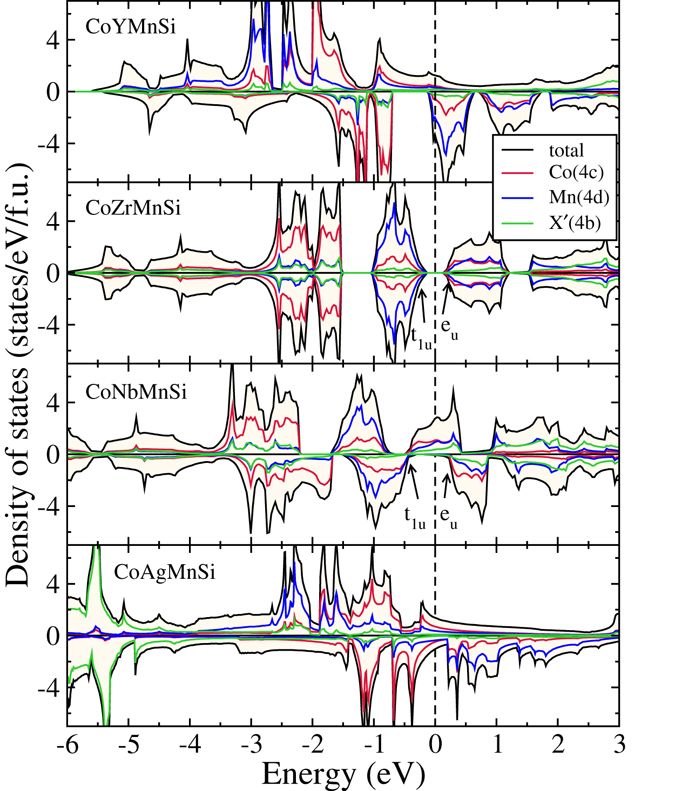
Spin polarized total and atom-projected densities of states for CoX′MnSi (X′ = Y, Zr, Nb, Ag) alloys. The ground states of these compounds are Type-I.

**Figure 5:**
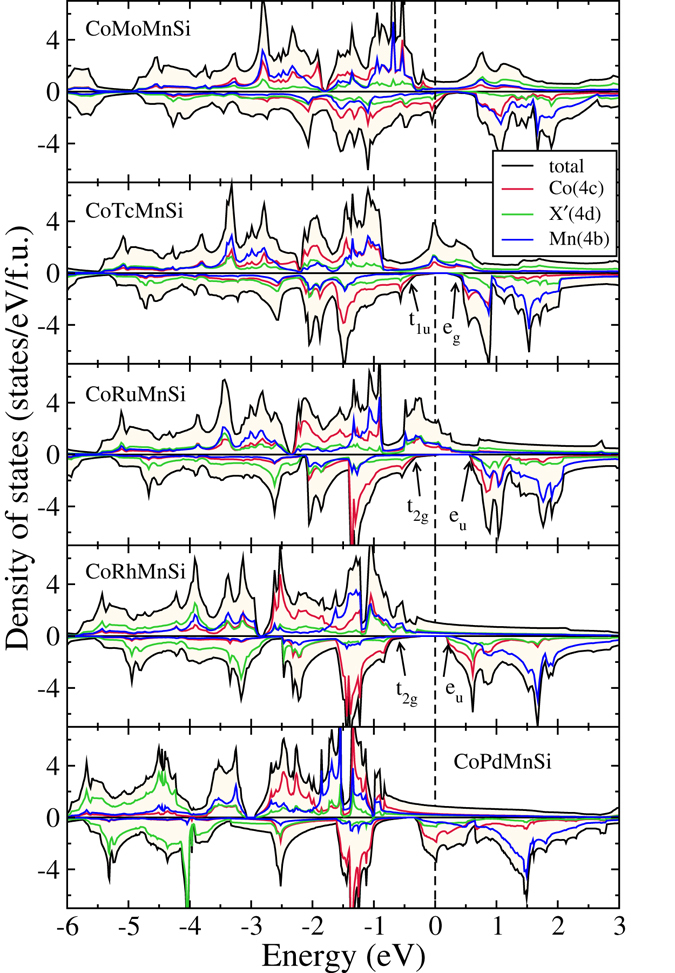
Spin polarized total and atom-projected densities of states for CoX′MnSi (X′ = Mo, Tc, Ru, Rh, Pd) alloys. The ground states of these compounds are Type-II.

**Figure 6:**
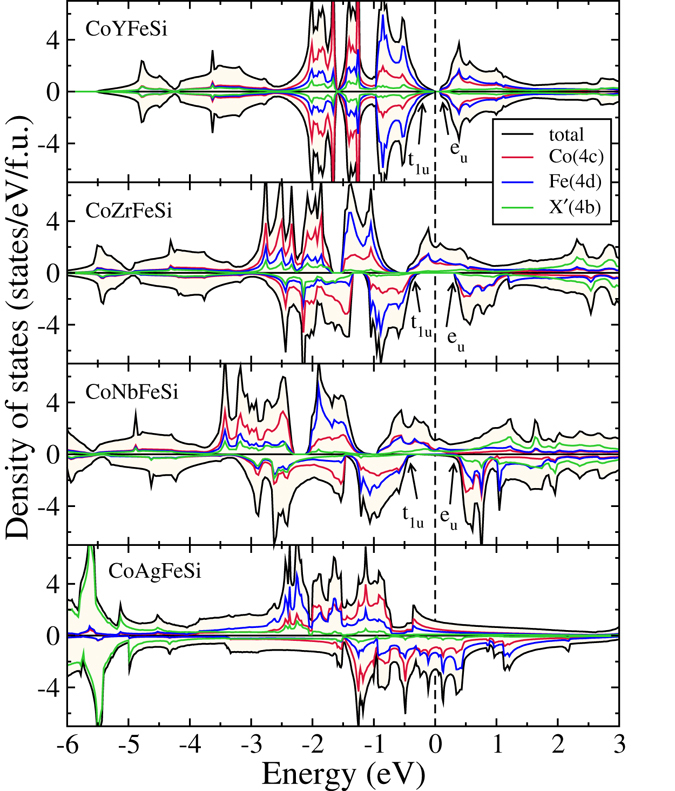
Spin polarized total and atom-projected densities of states for CoX′FeSi (X′ = Y, Zr, Nb, Ag) alloys. The ground states of these compounds are Type-I.

**Figure 7:**
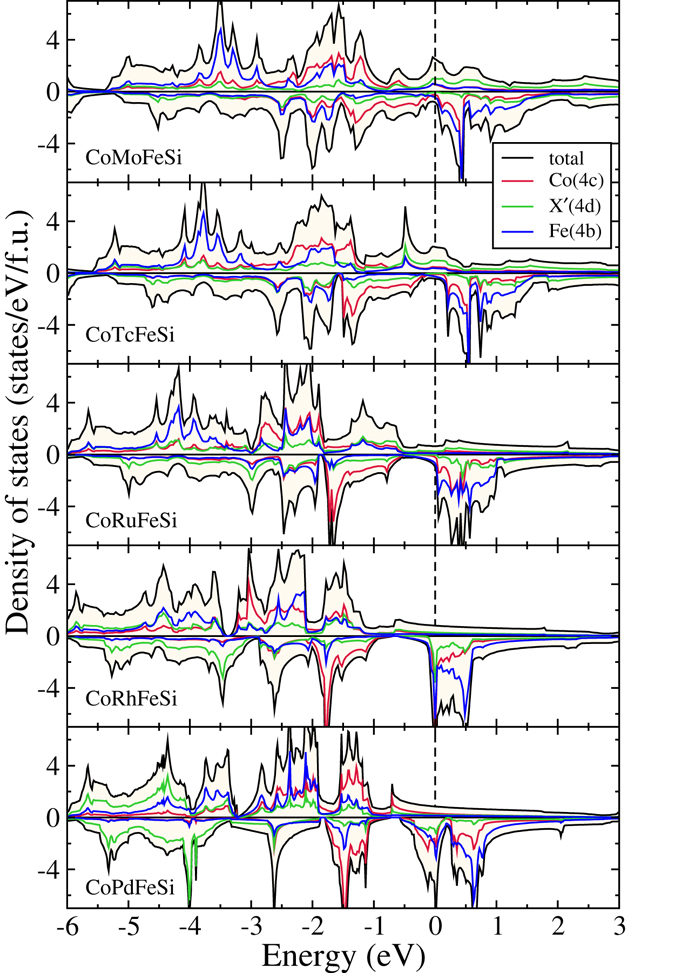
Spin polarized total and atom-projected densities of states for CoX′FeSi (X′ = Mo, Tc, Ru, Rh, Pd) compounds. The ground states of these compounds are Type-II.

**Figure 8:**
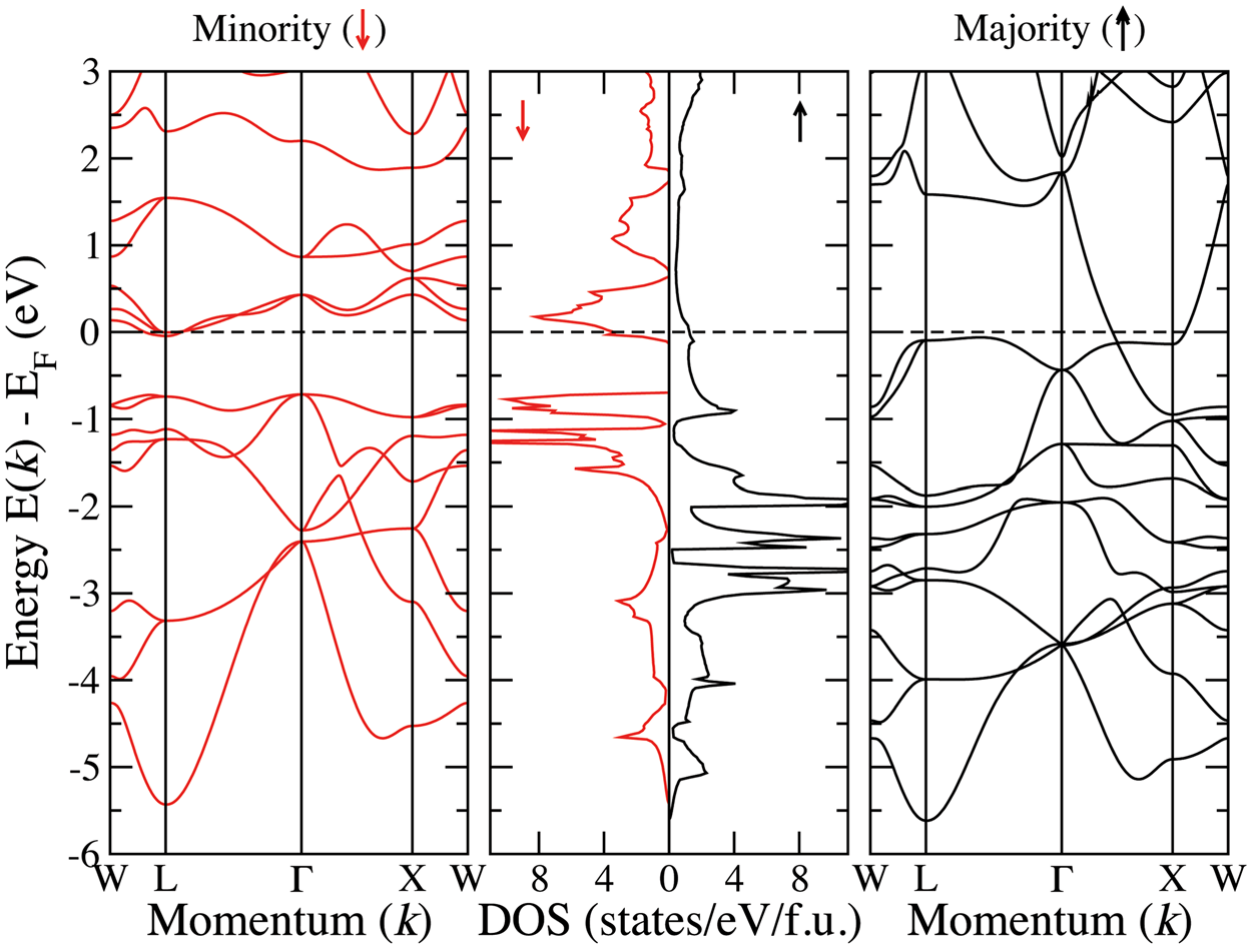
Spin-resolved band structure and density of states for CoYMnSi.

**Figure 9:**
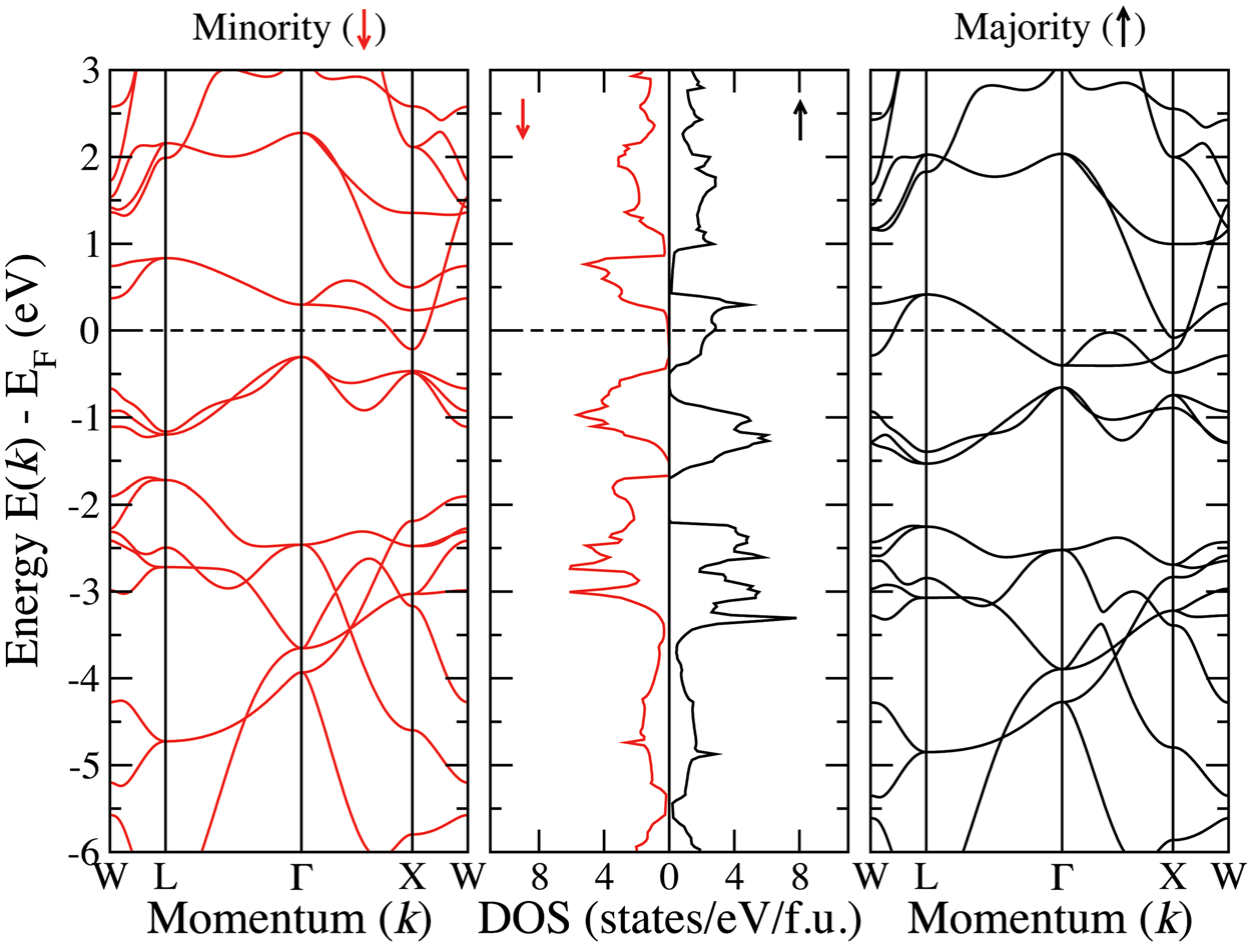
Spin-resolved band structure and density of states for CoNbMnSi.

The densities of states of CoX′Y′Si compounds in their respective ground state structures are presented in Figs 4, 5, 6 and 7. In Fig. 4, we show the densities of states of four CoX′MnSi systems, which crystallise in structure Type-I. We find out that CoYMnSi is distinctly different from the other compounds with the same structure. In CoYMnSi, the spin up bands are nearly full along with a large gap in the spin down band but the Fermi level does not fall into the gap. Upon analysis of the fat bands (not shown here), we find that the origin of the gap in the spin down band is due to the splitting of bonding *t*_2*g*_ (due to hybridisation between all transition metals) and nonbonding *t*_1*u*_ coming from Co and Mn. The separations between the bonding *t*_2*g*_ and nonbonding *t*_1*u*_ states are not enough to extend the gap till the Fermi level and lead to half-metallic behaviour. It may be noted that these features have been observed in ternary X_2_Y′Z compounds following *M* = *N*_*ν*_ − 18 Slater-Pauling rule^39^ and crystallising in Inverse Heusler structures. Thus, even with a quaternary compound having a 3*d* element replaced with a 4*d* element, the origin of the gap in the spin down band remains intact. The band structure of this compound presented in Fig. 8 shows that the minority bands cross the Fermi level at the *L* point. If the states crossing the Fermi level can be pushed into the higher energy region by adjusting the lattice constant or by doping with another element so that the total number of electrons is reduced, a half-metallic gap can be opened in this compound. CoZrMnSi having exactly 24 electrons, opens a semiconducting gap due to well separated *e*_*u*_ and *t*_1*u*_ hybrids coming from the Co and Mn atoms. Once again, this pattern is in agreement with that in ternary X_2_Y′Z compounds in Inverse Heusler structure following the same Slater-Pauling rule. CoNbMnSi, with one extra electron than CoZrMnSi, fills one out of two *e*_*g*_ states in the spin up band. The covalent nature of Co-Nb and Nb-Mn bonds enables this extra electron to be shared between Co and Mn. Therefore, the *e*_*g*_ states near the Fermi level are contributed mainly by Co and Mn. Since no electrons are accommodated in the down spin band, the semiconducting gap in spin down band remains. However, a very close inspection of the band structure (Fig. 9) of this compound reveals that in the down spin channel, a band crosses the Fermi level only slightly at the *X* point. Thus, although the magnetic moment of this system is integer, in strict sense, this is not a half-metal but can be considered as near half-metal. A little change in the lattice constant or suitable doping may push these states to the higher energy level and bring out the half-metallicity.

**Figure 10:**
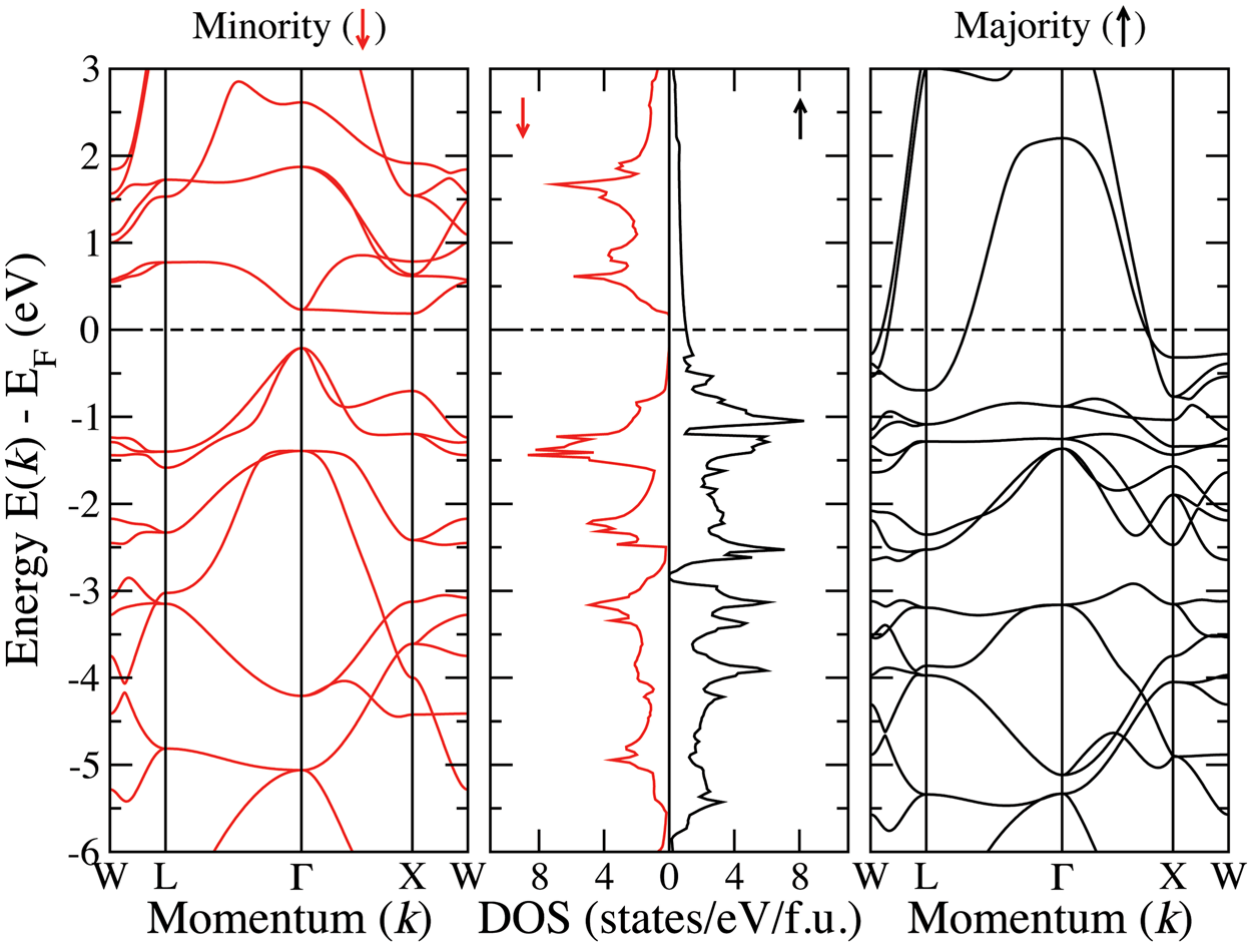
Spin-resolved band structure and density of states for CoRhMnSi.

In Fig. 5, we show the densities of states of five compounds that have Type-II as their ground state structures. Although CoNbMnSi and CoMoMnSi differ by only one electron, the trend observed from CoZrMnSi to CoNbMnSi is not followed when the compounds crystallise in a different structure. Substantial re-distribution of states near the Fermi level happens as one goes from CoNbMnSi to CoMoMnSi. We now see that the extra electron, in comparison to CoNbMnSi, is not solely accommodated in the spin up band of CoMoMnSi, resulting in spin down states at the Fermi level, followed by a gap. As the number of electron starts to increase when one goes from CoMoMnSi to CoRhMnSi, the extra electrons get accommodated in the spin up band, opening up a semiconducting gap at the spin down channel, and gradually filling up the spin up bands. As a result, CoTcMnSi, CoRuMnSi and CoRhMnSi exhibit half-metallicity with nearly 100% spin polarisation as is shown in Table 3. In order to establish that they indeed are half-metals (Unlike CoYMnSi and CoNbMnSi), we show the band structure of CoRhMnSi in Fig. 10. The origin of the half-metallicity in these compounds with structure Type-II is, however, do not reflect the established reason in case of ternary X_2_Y′Z compounds in regular Heusler structures^1^. Unlike Co_2_MnSi, in the present case, the Co and the Mn atoms sitting in different symmetry sites, have energy levels closer to each other. As a result the bonding *t*_2*g*_ hybrids originating from Co, Mn and X′ atoms and the non-bonding *t*_1*u*_ hybrids due to the Co and X′ atoms lie extremely close; same happens for anti-bonding *e*_*g*_ and non-bonding *e*_*u*_ states. As a result, the top of the spin down valence bands in case of CoMoMnSi and CoTcMnSi are due to the *t*_1*u*_ states while the bottom of the conduction bands are due to the *e*_*g*_ states. For CoRuMnSi and CoRhMnSi, they are due to the bonding *t*_2*g*_ and non-bonding *e*_*u*_ states respectively. The half-metallicity is destroyed in CoPdMnSi. Since the spin up is completely filled in CoRhMnSi, the extra electron in CoPdMnSi is accommodated in the spin down band, thus, pushing the *e*_*u*_ states towards lower energy and destroying half-metallic behaviour. The CoAgMnSi is significantly different as the *d* shell is completely filled in Ag, and the system crystallises in Type-I structure. The Ag states are now lying deep in energy. The Co and Mn hybridisations are weak, with a large spin polarisation of Mn states.

**Table 3:** Total and local magnetic moments in *μ*_*B*_/*f*.*u*. and the calculated spin polarization P of CoX′MnSi systems.

Systems (T_I_)	*N*_*ν*_	M	*M*_*Co*_	*M*_*Mn*_	*M*_*X*′_	*M*_*Si*_	P (%)
CoYMnSi	23	4.55	1.20	3.37	−0.09	0.00	46
CoZrMnSi	24	0.00	0.00	0.00	0.00	0.00	0
CoNbMnSi	25	0.99	0.71	0.30	0.00	0.00	96
**Systems (T**_**II**_ **)**	***N***_***ν***_	**M**	***M***_***Co***_	***M***_***X*′**_	***M***_***Mn***_	***M***_***Si***_	**P (%)**
CoMoMnSi	26	2.33	0.68	−0.58	2.27	−0.01	31
CoTcMnSi	27	3.00	0.67	−0.34	2.60	0.02	100
CoRuMnSi	28	4.00	0.92	0.13	2.94	−0.01	97
CoRhMnSi	29	5.00	1.21	0.43	3.33	−0.02	100
CoPdMnSi	30	4.90	1.11	0.21	3.47	−0.01	58
**Systems (T**_**I**_ **)**	***N***_***ν***_	**M**	***M***_***Co***_	***M***_***Mn***_	***M***_***X*′**_	***M***_***Si***_	**P (%)**
CoAgMnSi	31	3.53	0.49	3.08	0.02	0.08	14

*N*_*ν*_ is the number of valence electrons of the systems. M is the total magnetization.

Thus, in the CoX′MnSi series, we can identify one compound as nearly half-metal, CoNbMnSi and three compounds as half-metals, CoTcMnSi, CoRuMnSi and CoRhMnSi, with integer moments following Slater-Pauling rule and nearly 100% spin polarisation (Table 3). In Figs 6 and 7 we show the densities of states of CoX′FeSi compounds in Type-I and Type-II structures respectively. A comparison with the densities of states of CoX′MnSi compounds reveal that the features are very similar. CoYFeSi has 24 valence electrons and turns out to be a semiconductor with zero moment, with the gap in both spin bands coming out from separations in *t*_1*u*_ and *e*_*u*_ non bonding hybrids of Co and Fe. One extra electron in CoZrFeSi, like CoNbMnSi, occupies the spin up *e*_*u*_ states, leading to a semiconducting gap in the down spin channel. This compound, thus, turns out to be a half-metal with 100% spin polarisation (Tables 3 and 4). One more electron in CoNbFeSi fills up more states in the spin up band. However, though it seems like that the gap in the spin down band is intact, a close up view shows that few spin down states are there at the Fermi level. Thus the spin polarisation in this material is only 87%. None of the materials in Type-II structure qualify to be half-metals as all of them are characterised by the finite number of states at the Fermi level. As the number of electron increases while one goes from CoMoFeSi to CoRhFeSi, the extra electrons are accommodated in the spin down *e*_*u*_ bands, thus shifting the peak towards lower energy. For CoRhFeSi and CoPdFeSi, the peak in the spin down band falls exactly at the Fermi energy. This signifies structural instabilities in these compounds and may lead to ground state structures of different symmetries. This qualitative difference with respect to CoX′MnSi compounds is due to the extra electron of Fe in comparison to Mn. Since, like CoX′MnSi compounds in Type-II structure, the spin up bands in CoX′FeSi bands get filled, the extra electron in Fe is to be accommodated in the spin down band, leading to filling up states in the gap, and subsequent loss of possible half-metallicity.

**Table 4:** Total and atomic magnetic moments in *μ*_*B*_/*f*.*u*. and the calculated spin polarization P of CoX′FeSi systems.

Systems (T_I_)	*N*_*ν*_	M	*M*_*Co*_	*M*_*Fe*_	*M*_*X*′_	*M*_*Si*_	P (%)
CoYFeSi	24	0.00	0.00	0.00	0.00	0.00	0
CoZrFeSi	25	1.00	0.56	0.62	−0.12	0.00	100
CoNbFeSi	26	1.99	1.12	0.99	−0.06	0.02	87
**Systems (T**_**II**_ **)**	***N***_***ν***_	**M**	***M***_***Co***_	***M***_***X*′**_	***M***_***Fe***_	***M***_***Si***_	**P (%)**
CoMoFeSi	27	2.83	0.84	−0.33	2.31	0.04	59
CoTcFeSi	28	3.89	1.01	0.15	2.68	0.03	64
CoRuFeSi	29	4.66	1.26	0.53	2.87	0.01	87
CoRhFeSi	30	4.90	1.44	0.57	2.87	0.01	89
CoPdFeSi	31	4.00	1.08	0.07	2.84	−0.01	73
**Systems (T**_**I**_ **)**	***N***_***ν***_	**M**	***M***_***Co***_	***M***_***Fe***_	***M***_***X*′**_	***M***_***Si***_	**P (%)**
CoAgFeSi	32	2.71	0.69	2.14	0.03	−0.06	41

*N*_*ν*_ is the number of valence electrons of the systems. M is the total magnetization.

Thus, in the CoX′FeSi series, we find only CoZrFeSi to be half-metallic with integer magnetic moment, in accordance with Slater-Pauling rule, and high value of polarisation. CoNbFeSi could be considered nearly half-metal. Suitable doping with an element which reduces the total number of electrons may push the states at the Fermi level towards higher energies and bring in a complete spin polarisation in this compound. Among the compounds in Type-II structure, CoRuFeSi and CoRhFeSi have spin polarisations close to 90%. Experimentally, CoRuFeSi has been claimed to be a half-metal^14^, however through indirect evidence. This discrepancy between theory and experiment could be resolved by more direct experimental evidences like measurement of the spin polarization.

#### Trends in the local magnetic moments

In order to understand the trends in the total magnetic moments and the features in the densities of states, we look into the trends in the local magnetic moments associated with each species across the series studied. In Tables 3 and 4, results for total and the site projected magnetic moments for all the compounds in the CoX′MnSi and CoX′FeSi series, respectively, are presented. We find that the trend in the local magnetic moments is same for compounds irrespective of the series, as long as the structure type is same. The compounds having Type-I arrangement as their ground states and having valence electrons equal to or more than 24 follow the same trend in both series: both Co and Y′ element have low moments with Y′ having a vanishingly small moment. As number of electrons increase due to a different X′, the moments start to increase. The low exchange-splitting of the stronger magnet Y′ in both series can be understood from the neighbourhood around Y′ in Type-I structure. In Type-I structure, nearly non-magnetic 4*d* elements and Si form the neighbourhood around the Y′ element. This weakens the exchange field associated with the Y′ element and prevents building up of local moment in Y′. When an extra electron is added to the system due to a different 4*d* element, the covalent bonding between Co and Y′ makes them share the electron, resulting in nearly uniform increase in spin polarisations of both Co and Y′. The notable differences to this trend are found in cases of CoYMnSi, CoAgMnSi and CoAgFeSi, where both Mn and Fe have large spin polarisations. In these cases, the lattice constants of the systems are significantly larger in comparison to the other compounds with the same structure. Due to this, the Y′ atoms hybridise very little with the other atoms, thus, almost retaining their atomic moments. The most suitable example of this is CoYMnSi where the lattice constant is the largest and the Mn atom has a moment greater than 3 *μ*_B_. In case of systems with Type-II arrangement, Co and Y′ are nearest neighbours. The strong exchange field associated with them leads to large exchange splittings in the Y′ components. This gives rise to sizeable magnetic moments on Y′ and Co sites. The local magnetic moments systematically increases as the total number of electrons increase with change of X′ element. The changes in Fe moment is small compared to that in Mn as the number of electrons increase. This indicates that the Fe moments are more localised than the Mn moments. This, in turn, implies that the effects of localisation are to be included for compounds having Fe as has been mentioned earlier. Due to the strong exchange fields in the Type-II structures, the 4*d* elements too get slightly polarised.

#### Exchange coupling and Curie temperature

**Figure 11:**
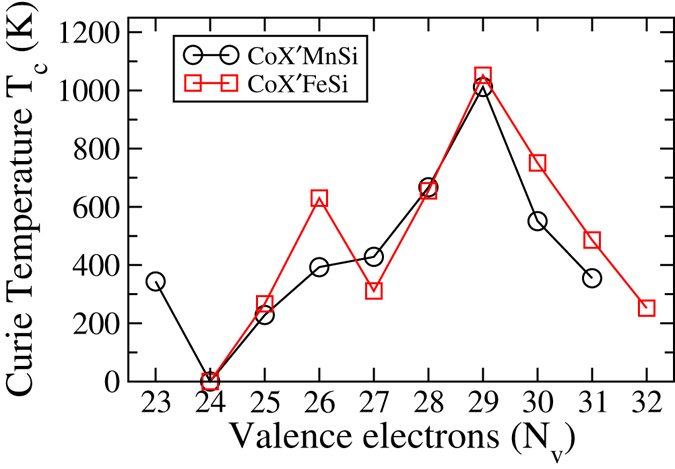
Calculated Curie temperatures with total number of valence electrons for CoX′Y′Si series.

In this section, we present the results on inter atomic effective exchange coupling constants (J_*eff*_) and Curie temperatures for CoX′Y′Si compounds in both series. The variations of the Curie temperatures with different X′ atoms i.e. with changes in the valence electron numbers are shown in Fig. 11. We find two compounds, CoRhMnSi and CoRuFeSi, having very high Curie temperatures, 1012 K and 1052 K respectively. The experimental Curie temperature for CoRuFeSi is 867 K^14^. Our results are overestimated in comparison to the experimental one as we have used mean field approximation. Apart from these two, there are few other compounds with Curie temperatures between 600 K and 800 K (Fig. 11). The results show that the variations in the Curie temperatures can be classified in two distinct regions based on the structure types in which the compounds crystallise. In each region, however, the variations are not uniform. This is true for the two different series also. For Type-I structures, the Curie temperatures are low. For Type-II structures they increase with *N*_*ν*_ steadily upto a critical point *N*_*ν*_ = 29 after which it steadily decreases. The maximum Curie temperature in both CoX′MnSi and CoX′FeSi are, therefore, obtained when *N*_*ν*_ = 29.

**Figure 12:**
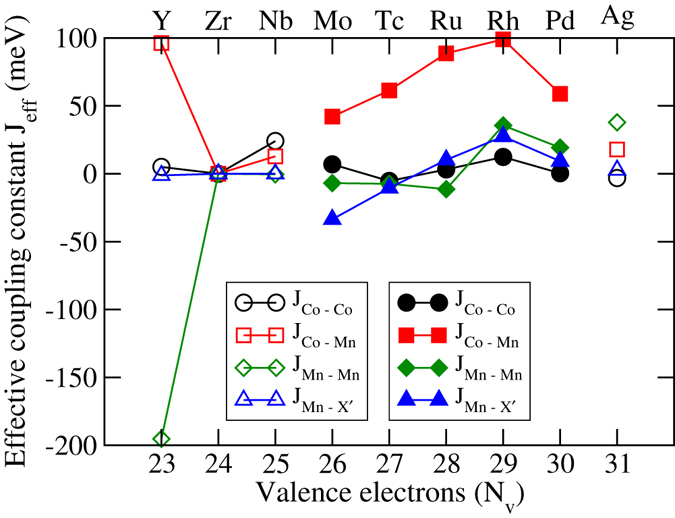
Effective exchange coupling constants for CoX′MnSi alloys. Open symbols: Type-I structure, Filled symbols: Type-II structure.

**Figure 13:**
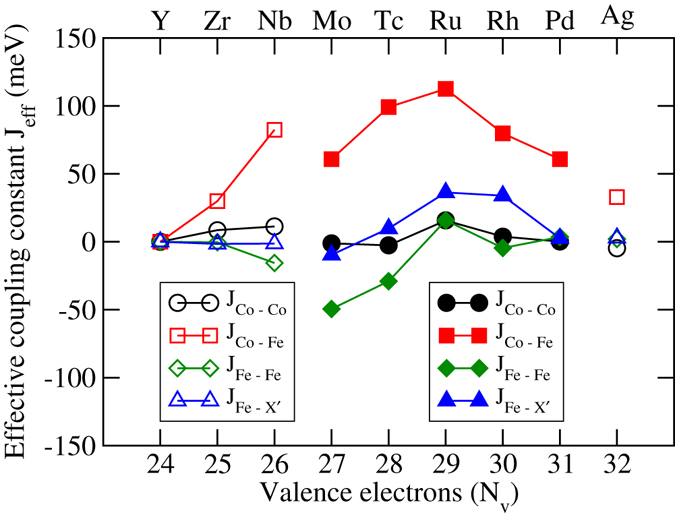
Effective exchange coupling constants for CoX′FeSi alloys. Open symbols: Type-I structure, Filled symbols: Type-II structure.

In order to explain such behaviour of Curie temperatures, we take recourse to the calculated inter-atomic magnetic exchange coupling parameters, and the features in the densities of states. In Figs 12 and 13, we show the various effective exchange coupling parameters (J_*eff*_). We find that the Co-Co, Co-Y′ and Y′-Y′ effective exchange parameters are the dominant ones. The other exchange interactions are very weak and do not contribute to the understanding of the trends in the Curie temperature. Analysing the results for exchange interactions in compounds with two different structure types, we see that Co-Y′ interaction is the dominant one irrespective of the structure type. In general, the Co-Y′ effective exchange parameters for compounds in structure Type-II are significantly larger than for those in structure Type-I. The weaker Co-Y′ interactions for compounds in structure Type-I are due to the fact that Co and Y′ interactions are mediated via the weak magnet X′ in structure Type-I while the interactions are direct in structure Type-II. A notable exception to this trend is found in case of CoNbFeSi which has structure Type-I as the ground state but the Co-Y′ exchange parameter is larger than that in CoMoFeSi, the first compound in the series having structure Type-II as the ground state. That the variations in the Co-Y′ exchange parameter decides the variations in the Curie temperature is visible upon comparing the trends of both quantities. Thus, a stronger Co-Y′ exchange parameter in CoNbFeSi leads to a higher Curie temperature in comparison to CoMoFeSi, in spite of the former crystallising in structure Type-I and thus, having an indirect Co-Y′ exchange. The other exchange parameters have similar variations qualitatively across the two series, and they only contribute to the absolute values of the Curie temperatures. The prominent ones except Co-Y′ are the X′-Y′ and the intra-atomic Y′-Y′ interactions. The exceptional behaviour of CoYMnSi in the context of total magnetic moment is also observed here. In case of this compound, the Co-Mn interaction strength is of the same order as that of CoRhMnSi, the compound having the maximum value of Curie temperature in the series. However, this strong ferromagnetic Co-Y′ interaction is completely compensated by a stronger Mn-Mn interaction, which is antiferromagnetic in nature, leading to a smaller value of Curie temperature.

The result on Curie temperatures revel another interesting fact. We find that the half-metals with 100% spin polarizations having structure Type-II have high Curie temperatures (428 K for CoTcMnSi and 1012 K for CoRhMnSi) while the only half-metal with 100% spin polarization having structure Type-I, CoZrFeSi, has a rather low Curie temperature of 267 K. This suggests that the half-metallicity cannot be always correlated with occurrence of high Curie temperature. As the discussion in the preceding paragraph suggest, the magnitude of Curie temperature is decided by the select exchange interaction and their magnitudes, which in turn are dependent upon the structure types. Thus, the crystal structure plays the most important role influencing the magnitude of the Curie temperatures.

The question that remains to be answered is that why the *J*_*eff*_ values have the maximum at *N*_*ν*_ = 29 followed by a decrease upon increase in *N*_*ν*_. Kubler *et al*.^40^, in their investigations of the ternary Co_2_Y′Z compounds found a similar behaviour in the variations of the Curie temperatures with *N*_*ν*_. They attributed this to the changes in the behaviour of the “Exchange average”, the average of exchange energies associated with low temperature spin excitations. This variation in the “Exchange average” is related to the availability of spin down states below Fermi level. They showed that a gap in the spin down bands starting below Fermi level and extending beyond it would lead to a larger value of “Exchange average” and consequently a larger Curie temperature. Extending this idea to the compounds in the present work, we find that the unavailability of the states in the spin down bands gradually increases as *N*_*ν*_ increases and is maximised at *N*_*ν*_ = 29. Thus the “Exchange average” is maximised at *N*_*ν*_ = 29 and we see its manifestations in the maximisations of the *J*_*eff*_. Upon further increase of *N*_*ν*_, we see states in the spin down bands at the Fermi level. This reduces the “Exchange average” and the *J*_*eff*_ and Curie temperatures decrease beyond *N*_*ν*_ = 29.

## Discussion

We have made a detailed investigations into the structural, electronic and magnetic properties of 18 Quaternary Heusler compounds spanning the series CoX′Y′Si, where X′ stand for 9 elements with 4*d* electrons in their valence shells, and Y′ being Fe and Mn, the two strong magnets with unfilled 3*d* electrons in their outer shells. Our search for new half-metallic ferromagnets from quaternary series with both 3*d* and 4*d* electrons in the same compound throws up interesting facts. We find that although there are seven compounds with integer values of their magnetic moments, all of them do not qualify to be half-metals or even near half-metals. It has been found that three compounds CoTcMnSi, CoRhMnSi and CoZrFeSi can be identified as truly half-metals. However, there are few other compounds like CoYMnSi, CoNbMnSi, CoRuMnSi and CoNbFeSi, which can be identified as near half-metals. Application of moderate pressure or controlled doping in a way that the total number of electrons in these systems reduce, should make these systems half-metallic too. In the CoX′FeSi series, we do not get many candidates with half-metallic behaviour but their electronic structures suggest that half-metallicity too can be induced in these compounds by application of moderate pressure, which will shift the Fermi levels to a gap in the spin down bands. We provide an understanding of the types of site occupancies observed in the compounds of the two series in terms of relative values of electronegativities of Co, X′ and Y′. This explanation towards the preferred structure types of quaternary Heusler compounds addresses ground state structure types of large numbers of compounds in the quaternary family outside of the two series studied in this work. The trends in the magnetic properties and the electronic structures of these compounds are found to be due to the neighbourhood of the magnetic atoms, which differ between the two preferred structure types. Consequently, the magnetic interactions between the stronger magnetic elements, Co and Y′, get modified as the structure type or the element X′ changes. We find that the magnetic exchange interactions between Co and Y′ atoms are the strongest and their behaviour with number of valence electrons is responsible for the trends in the Curie temperature, another quantity of interest for assessing a half-metal in the context of its potential for spintronics applications. We make an assessment of the variations in the magnetic exchange averages across the compounds from the features in their densities of states and conclude that the variations of the Curie temperatures can be understood from it. The compounds crystallising in Type-I structures have lower Curie temperatures due to weaker exchange interactions between the constituents, making the average exchange lower. The compounds with structure Type-II have higher Curie temperature due to stronger Co-Y′ exchange interactions which make the average exchange stronger. In both series we find that the maximum Curie temperature is achieved for compounds with *N*_*ν*_ = 29, and in general high values of Curie temperatures are obtained if the number of valence electrons are in a range of 28–30. Accordingly, out of the compounds identified as half-metals in the present study, CoTcMnSi and CoRhMnSi have significantly high values of Curie temperature and hence these two can be considered as new functional materials for magnetic applications. If CoNbFeSi, CoTcFeSi, CoRuFeSi and CoRhFeSi turn out to be half-metals under moderate pressure, they also would fall into this category as they too have high values of Curie temperature. Overall, this work, apart from being able to find new materials for spintronics applications from Quaternary Heusler series with 3*d*–4*d* combinations, has addressed the fundamental issues related to the understanding of the trends in the structural, electronic and magnetic properties of these compounds.

## Methods

Electronic structure calculations are performed with spin-polarized density functional theory (DFT) based projector augmented wave method as implemented in Vienna Ab-initio Simulation Package (VASP)^41–43^. The exchange and correlation part of the Hamiltonian is described by Generalised Gradient Approximation (GGA)^44^. For self consistent calculations, we have used an energy cut-off of 450 eV and a Monkhorst-Pack^45^ 25 × 25 × 25 k-mesh. A larger k-mesh of 31 × 31 × 31 was used for calculations of the densities of states. The energy convergence and the force convergence criteria were set to ${10}^{-6}$ eV and ${10}^{-2}$ eV/Å respectively. Spin-orbit coupling was not included in the calculations.

The magnetic pair exchange parameters have been calculated with multiple scattering Green function formalism as implemented in SPRKKR code^46^. In here, the spin part of the Hamiltonian is mapped to a Heisenberg model

1 $$H=-\,\sum _{\mu ,\nu }\sum _{i,j}{J}_{ij}^{\mu \nu }{{\bf{e}}}_{i}^{\mu }\mathrm{.}{{\bf{e}}}_{j}^{\nu }$$

*μ*, *ν* represent different sub-lattices, *i*, *j* represent atomic positions and ${{\bf{e}}}_{i}^{\mu }$ denotes the unit vector along the direction of magnetic moments at site *i* belonging to sub-lattice *μ*. The ${J}_{ij}^{\mu \nu }$s are calculated from the energy differences due to infinitesimally small orientations of a pair of spins within the formulation of Liechtenstein *et al*.^47^. In order to calculate the energy differences by the SPRKKR code, full potential spin polarized scaler relativistic Hamiltonian with angular momentum cut-off *l*_*max*_ = 3 is used along with a uniform k-mesh of 22 × 22 × 22 for Brillouin zone integrations. The Green’s functions were calculated for 32 complex energy points distributed on a semicircular contour. The energy convergence criterion was set to 10^−5^ eV for the self-consistence cycles. Equilibrium lattice parameters obtained from the projector augmented wave method have been used throughout. Once the ${J}_{ij}^{\mu \nu }$s are calculated, they were used further to compute the Curie temperatures within the mean field approximation^48^ for which ${k}_{B}{T}_{c}=\frac{2}{3}{J}_{max}$; *J*_*max*_ is the largest eigenvalue of the ${J}_{eff}^{\mu \nu }$ matrix with ${J}_{eff}^{\mu \nu }$, the effective exchange coupling constant being given as ${J}_{eff}^{\mu \nu }={\sum }_{j}{J}_{oj}^{\mu \nu }$; *0* being fixed within the *μ* sublattice and *j* runs over *ν* sublattice.
